# Identification of vascular endothelial growth factor in preeclampsia in Iraqi women

**DOI:** 10.25122/jml-2021-0211

**Published:** 2022-10

**Authors:** Basima Al-Ghazali, Muna Khadim, Sara Hamza, Hawraa Sahib Al-Haddad, Alaa Salah Jumaah, Najah Hadi

**Affiliations:** 1 Department of Gynecology and Obstetrics, Faculty of Medicine, University of Kufa, Kufa, Iraq; 2 Department of Obstetrics and Gynecology, Al-Furat Al-Awsat Hospital, Kufa, Iraq; 3 High Institute for Infertility Diagnosis and Assisted Reproductive Technologies, Baghdad, Iraq; 4 Department of Pathology and Forensic Medicine, Faculty of Medicine, University of Kufa, Kufa, Iraq; 5 Department of Pharmacology and Therapeutics, Faculty of Medicine, University of Kufa, Najaf, Iraq

**Keywords:** vascular endothelial growth factor, preeclampsia, growth factor in preeclampsia

## Abstract

Preeclampsia (PE) is a major obstetric syndrome and represents a pregnancy hypertensive disease affecting about 2–8% of pregnancies. Typically, it occurs after 20 weeks of pregnancy, being classified as early or late in accordance with the gestational age at diagnosis or delivery. An imbalance between angiogenic and antiangiogenic factors has an important role in the pathophysiology of PE. It was hypothesized that the dysfunctional endothelium contributes to the pathogenesis of PE. A change in the production of Vascular endothelial growth factor (VEGR), a biomarker of endothelial dysfunction, is associated with this disease, whether presenting an increase, decrease, or being at a normal level. This study examined the associations between VEGF and preeclampsia and the importance of this VEGF as a predictor of its severity. This case-control study included 50 patients with preeclampsia and 50 normotensive pregnant women in the control group. Venous blood was aspirated from each patient, and VEGF levels were measured from sera. The mean VEGF for patients with mild PE was 29.410±18.976 pg/ml, for those with severe PE it was 36.188±36.98 pg/ml, and for normotensive women it was 92.104±154.715 pg/ml. There were significant differences in VEGF levels between the studied groups (P=0.024). This study showed that serum VEGF levels were significantly reduced in patients with preeclampsia compared with normotensive pregnant women, suggesting marked endothelial dysfunction. This led to widespread vasoconstriction and, in turn, caused hypertension and proteinuria.

## INTRODUCTION

Preeclampsia (PE) is a major obstetric syndrome [1] and is a pregnancy hypertensive disease affecting about 2–8% of pregnancies. Typically it occurs after 20 weeks of pregnancy. The diagnosis of preeclampsia requires a systolic blood pressure ≥140 mmHg or a diastolic blood pressure ≥90 mmHg plus one or more of the followings: thrombocytopenia, proteinuria, liver function impairment, renal insufficiency, cerebral or pulmonary edema, visual disturbances. Severe preeclampsia usually occurs before the 20^th^ week of gestation and with systolic blood pressure ≥160mmHg [2, 3]. The syndrome is classified as early or late in accordance with gestational age at diagnosis or delivery. Early PE is associated with more complications, such as multisystem involvement and placental vascular lesion due to hypoperfusion [4]. It was proposed that an imbalance between angiogenic and antiangiogenic factors has an important role in the pathophysiology of PE [4]. Alterations on the concentrations of angiogenic factors, placental growth factor (PIGF), soluble vascular endothelial growth factor receptor-1 (sVEFGR-1), known as fms-like tyrosine kinase -1 (sflt-1) in maternal circulation may precede preeclampsia [5, 6]. Despite extensive research, the exact cause of preeclampsia is still unknown [7, 8]. It was hypothesized that dysfunctional endothelium contributes to the pathogenesis of PE. Changes in the production of vascular endothelial growth factor (VEGF), a biomarker of endothelial dysfunction, are associated with this disease whether the production increases, decreases, or remains at a normal level [9, 10]. It was believed that PE is associated with changes in maternal VEGF plasma concentrations and other growth factors. Vascular endothelial growth factor was thought to be implicated in the pathogenesis of PE due to its role in the physiology of vasculogenesis and vascular permeability [10]. VEGF increased the production of nitric oxide, which was believed to be a strong vasodilator in normal gestation [11]. Sufficient knowledge regarding the role of VEGF in PE is important in order to design a paradigm for a screening strategy targeting the early detection and treatment of this disease. Many studies try to investigate the possible role of these angiogenic factors in the pathogenesis of PE. However, results remain elusive and conflicting [6, 12–19]. This study investigated the associations between VEGF and preeclampsia and its importance as a predictor of disease severity.

## MATERIAL AND METHODS

This case-control study was performed in the Al-Zahraa teaching hospital for maternity and pediatrics and included 50 patients with preeclampsia and 50 normotensive pregnant women as the control group. Data was collected on the following variables: maternal age, parity, blood pressure, urinary protein, complete blood count, renal function test and liver function test. Preeclampsia was diagnosed as follows: systolic blood pressure ≥140 mmHg or diastolic blood pressure ≥90 mmHg with proteinuria (2-4++) on dipstick test for patients at more than 20 weeks of gestation, provided that the patient was previously normotensive. Diabetic patients, patients with chronic renal diseases, and chronic hypertensive women were excluded from this study. Venous blood was aspirated from each patient, and VEGF levels were measured from sera using human VEGF (NBPI-91272) by ELISA (enzyme-linked immunoassay method). Statistical analysis was performed using the analysis of variance (ANOVA) test at ≤0.05 significance level. All statistical procedures were done by SPSS version 23 and Microsoft Excel 2013.

## RESULTS

The research included 50 pregnant women with preeclampsia with an age range of 19–30 years for all groups, as well as 50 normotensive pregnant women as a control group. Laboratory results of each group are shown in Table 1.

**Table 1:** Demographic data and laboratory results of patients enrolled in this study.

Patient characteristics	Normal	Mild PE	Severe PE	P-value
**Number**	50	14	36	
**Mean age**	23.940±2.860	25.780±2.900	23.940±2.860	0.890
**Systolic blood pressure**	119.060±17.281	145.200±2.804	178.270±8.669	0.000
**Diastolic blood pressure**	77.780±5.366	94.142±2.801	115.250±3.574	0.000
**Proteinuria g/l**	0	1.571±0.872	3.583±2.654	0.000
**Blood urea**	26.260±5.170	26±3.390	46.277±8.701	0.000
**Serum creatinine mg/dl**	0.667±0.234	0.700±0.252	1.616±0.311	0.000
**Serum GPT**	21.880±7.370	24.285±6.687	39.628±4.943	0.000
**Serum GOT**	27.440±6.583	26.285±7.122	41.722±11.007	0.000
Platelet count *(10^3^)	256.840±55.179	198.928±14.498	122.250±14.952	0.617

PE – Preeclampsia; Serum GPT – Serum Glutamic Pyruvic Transaminase; Serum GOT – Serum Glutamic-Oxaloacetic Transaminase.

**Figure 1:**
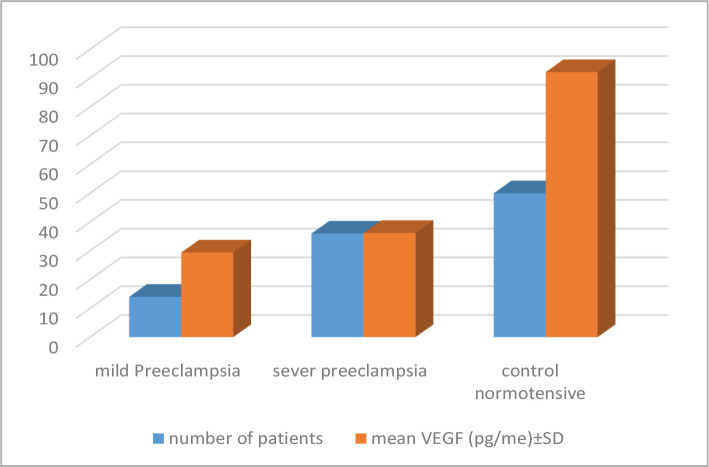
Mean VEGF in the studied groups.

Fourteen patients with a mean age of 25.780±2.900 years had mild PE, while 36 patients with a mean age of 23.940±2.860 years had severe PE. There were 50 pregnant normotensive women with a mean age of 23.940±2.860 years in the control group (Table 1). The mean VEGF for patients with mild PE was 29.410±18.976 pg/ml; for those with severe PE, it was 36.188±36.98 pg/ml, and for normotensive women, it was 92.104±154.715 pg/ml. There were significant differences in the VEGF level between the studied groups (P=0.024), as presented in Table 2 and Figure 1.

**Table 2:** Mean VEGF in the studied groups.

Groups	Number of patients	Mean VEGF (pg/me)±SD
Mild preeclampsia	14	29.410±18.976
Severe preeclampsia	36	36.188±36.890
Control normotensive	50	92.104±154.715

* – P-value=0.0242.

**Figure 2:**
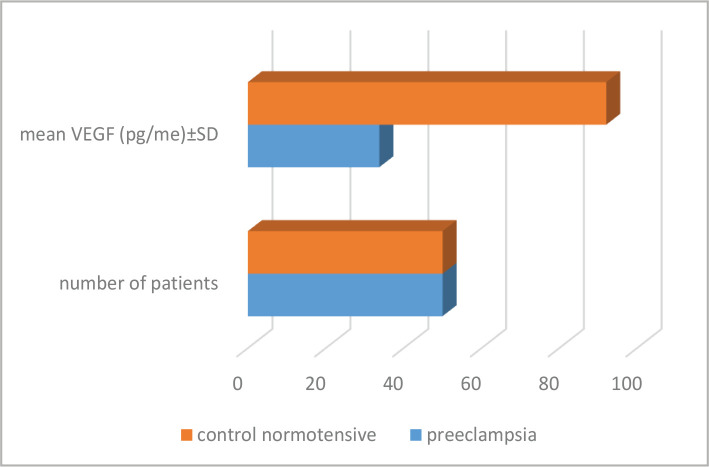
VEGF in patients with PE and normotensive women.

The serum level of VEGF was significantly higher (92.104±154.715 pg/ml) in normotensive pregnant women (control group) than in patients with PE (33.76±35.298 pg/ml) (p=0.011) (Table 3 and Figure 2).

**Table 3:** Serum VEGF in patients with PE and normotensive pregnant women.

Groups	Number of patients	Mean VEGF (pg/me)±SD
Preeclampsia	50	33.76±35.298
Control normotensive	50	92.104±154.715

* – P-value=0.011.

There were no significant differences in VEGF serum levels between patients with severe PE and those with mild PE (P-value=0.519) (Table 4).

**Table 4:** Serum VEGF in patients with severe and mild PE.

Groups	Number of patients	Mean VEGF (pg/me)±SD
Mild preeclampsia	14	29.410±18.976
Sever preeclampsia	36	36.188±36.890

* – P-value=0.519.

## DISCUSSION

The possible contribution of VEGF in the pathophysiology of preeclampsia has been extensively studied; however, the results of previous studies are conflicting [9, 10, 20]. This study showed that the serum level of VEGF was significantly reduced in patients with preeclampsia compared with normotensive pregnant women (p<0.05). The results of our study were supported by the results of other studies [21–25]. The reduction of serum VEGF in preeclampsia could be caused by high levels of sflt-1 (soluble fms-like tyrosine kinase-1). Sflt-1 binds and inhibits several proangiogenic proteins such as placental growth factor (PIGF) and VEGF. This inhibition occurs by preventing the communication of these angiogenic factors with their receptors [26]. The physiologic action of VEGF is mediated by binding into two high-affinity tyrosine kinase receptors. Vascular endothelial cells selectively expressed these receptors [9, 20–23]. Soluble flt-1, a splice variant of flt-1, represents a potent antagonist. This antagonistic effect is mediated by binding to VEGF and inhibiting its biological activity [26]. A high level of soluble flt-1 was secreted in severe preeclampsia, which neutralized the effect of PIGF and VEGF [27]. A marked reduction of VEGF in preeclampsia denotes severe endothelial dysfunction and dysregulation, manifested as severe hypertension and proteinuria. A high level of flt-1 was associated with the level of VEGF and severe endothelial dysfunctions [28], leading to severe clinical features. In our study, we did not estimate serum flt-1 simultaneously with VEGF.

However, there was good evidence from other studies that demonstrated an increased level of flt-1 in preeclampsia, which led to decreased circulating free VEGF [28]. High levels of flt-1 are associated with glomerular endotheliosis, proteinuria, and hypertension in non-pregnant and pregnant rats, which are important features of preeclampsia [29]. This evidence suggests that low levels of VEGF lead to endothelial dysfunction, which was implicated in the pathogenesis of preeclampsia. In contrast, VEGF may be normal, increased, or decreased in uncomplicated normotensive pregnancy [20, 30, 31]. The lack of measurement of flt-1 concurrently with VEGF in this study is considered a limitation as this may provide a good insight into the possible interaction between these factors on damage to endothelial cells in patients with preeclampsia. Our study showed no significant differences in serum VEGF levels between patients with mild or severe preeclampsia, as presented in Table 3. These findings may be attributed to the fact that determining the severity of the disease on the clinical background may not reflect the extent of damage dysfunction occurring in endothelial cells. Based on the above observation, the clinical decision on the severity of preeclampsia is better to be based on clinical as well as monitoring biological markers. The conflicting reports regarding maternal vascular endothelial factor levels in patients with preeclampsia and in normotensive pregnant women could be attributed to the detection method and the date of VEGF measurement with respect to gestational age. Restoration of endothelial function may be achieved by administering exogenous VEGF [10]. The use of pro-angiogenic analogue might be beneficial in the management of preeclampsia by improving the function of the endothelium, while the use of nicotine may be beneficial in the management of preeclampsia as it improves angiogenesis, yet it is not advisable as it is associated with intrauterine growth retardation [32]. Meanwhile, a lower incidence of preeclampsia was shown in smoker women due to decreased serum levels of sflt-1 [33].

## CONCLUSION

This study showed that VEGF levels were significantly reduced in patients with preeclampsia compared with normotensive pregnant women, suggesting marked endothelial dysfunction. This led to widespread vasoconstriction, causing hypertension and proteinuria. However, serum VEGF was reduced in both mild and severe preeclampsia, suggesting the importance of VEGT in the pathogenesis of preeclampsia as a cause of endothelial dysfunction. Nonetheless, clinical evidence of severity may not reflect the true extent of endothelial damage and dysfunction.

## Personal thanks

The authors thank all who helped and encouraged the development of this research.

## References

[1] Tarca AL, Romero R, Benshalom-Tirosh N, Than NG (2019). The prediction of early preeclampsia: Results from a longitudinal proteomics study. PLoS One.

[2] Eddy AC, Bidwell GL, George EM (2018). Pro-angiogenic therapeutics for preeclampsia. Biol Sex Differ.

[3] Lambert G, Brichant JF, Hartstein G, Bonhomme V, Dewandre PY (2014). Preeclampsia: an update. Acta Anaesthesiol Belg.

[4] Chaiworapongsa T, Romero R, Korzeniewski SJ, Kusanovic JP (2013). Maternal plasma concentrations of angiogenic/antiangiogenic factors in the third trimester of pregnancy to identify the patient at risk for stillbirth at or near term and severe late preeclampsia. Am J Obstet Gynecol.

[5] Chaiworapongsa T, Romero R, Kim YM, Kim GJ (2005). Plasma soluble vascular endothelial growth factor receptor-1 concentration is elevated prior to the clinical diagnosis of preeclampsia. J Matern Fetal Neonatal Med.

[6] Park CW, Park JS, Shim SS, Jun JK (2005). An elevated maternal plasma, but not amniotic fluid, soluble fms-like tyrosine kinase-1 (sFlt-1) at the time of mid-trimester genetic amniocentesis is a risk factor for preeclampsia. Am J Obstet Gynecol.

[7] Agarwal I, Karumanchi SA (2011). Pre-eclampsia and the Anti-Angiogenic State. Pregnancy Hypertens.

[8] Wang A, Rana S, Karumanchi SA (2009). Pre-eclampsia: The Role of Angiogenic Factors in Its Pathogenesis. Physiology (Bethesda).

[9] Tsatsaris V, Goffin F, Munaut C, Franc J (2003). Over expression of the Soluble Vascular Endothelial Growth Factor Receptor in Pre-eclampsic Patients: Path physiological Consequences. J Clin Endocrinol Metab.

[10] Maynard SE, Min JY, Merchan J, Lim KH (2003). Excess placental soluble fms like tyrosine kinase 1 (sFlt1) may contribute to endothelial dysfunction, hypertension, and proteinuria in preeclampsia. J Clin Invest.

[11] Adu-Bonsaffoh K, Antwi DA, Gyan B, Obed SA (2017). Endothelial dysfunction in the pathogenesis of preeclampsia in Ghanaian women. BMC Physiol.

[12] Romero R, Chaiworapongsa T, Erez O, Tarca A (2010). An imbalance between angiogenic and antiangiogenic factors precedes fetal death in a subset of patients: results of a longitudinal study. J Matern Fetal Neonatal Med.

[13] Smith GC, Crossley JA, Aitken DA (2007). Circulating angiogenic factors in early pregnancy and the risk of preeclampsia, intrauterine growth restriction, spontaneous preterm birth, and stillbirth. Obstet Gynecol.

[14] Parra-Cordero M, Rodrigo R, Barja P, Bosco C (2012). Prediction of early and late preeclampsia from maternal characteristics, uterine artery Doppler and markers of Vasculogenesis during the first trimester of pregnancy. Ultrasound Obstet Gynecol.

[15] Di LG, Ceccarello M, Cecotti V, Ronfani L (2012). First trimester maternal serum PIGF, free beta-hCG, PAPP-A, PP-13, uterine artery Doppler and maternal history for the prediction of preeclampsia. Placenta.

[16] Poon LC, Akolekar R, Lachmann R, Beta J, Nicolaides KH (2010). Hypertensive disorders in pregnancy: screening by biophysical and biochemical markers at 11–13 weeks. Ultrasound Obstet Gynecol.

[17] Pedrosa AC, Matias A (2011). Screening for preeclampsia: a systematic review of tests combining uterine artery Doppler with other markers. J Perinat Med.

[18] Wikstrom AK, Larsson A, Eriksson UJ, Nash P (2007). Placental growth factor and soluble FMS-like tyrosine kinase-1 in early-onset and late onset preeclampsia. Obstet Gynecol.

[19] Robinson CJ, Johnson DD, Chang EY, Armstrong DM, Wang W (2006). Evaluation of placenta growth factor and soluble Fms-like tyrosine kinase 1 receptor levels in mild and severe preeclampsia. Am J Obstet Gynecol.

[20] George EM, Granger JP (2010). Recent insights into the pathophysiology of preeclampsia. Expert Rev Obstet Gynecol.

[21] Levine RJ, Maynard SE, Qian C, Lim KH (2004). Circulating angiogenic factors and the risk of preeclampsia. N Engl J Med.

[22] Sun SG, Shen N, Zheng YH, Shang T (2006). [Expression of hypoxia-inducible factor-1alpha, vascular endothelial growth factor and sFlt-1 in preeclampsia placenta]. Zhonghua Fu Chan Ke Za Zhi.

[23] Varughese B, Bhatla N, Kumar R, Dwivedi SN, Dhingra R (2010). Circulating angiogenic factors in pregnancies complicated by preeclampsia. Natl Med J India.

[24] Yelumalai S, Muniandy S, Omar SZ, Qvist R (2010). Pregnancy induced hypertension and preeclampsia: Levels of angiogenic factors in Malaysian women. J Clin Biochem Nutr.

[25] Masuyama H, Nakatsukasa H, Takamoto N, Hiramatsu Y (2007). Correlation between Soluble Endoglin, Vascular Endothelial Growth Factor Receptor-1, and Adipocytokines in Pre-eclampsia. J Clin Endocrinol Metab.

[26] George EM, Granger JP (2010). Recent insights into the pathophysiology of preeclampsia. Expert Rev Obstet Gynecol.

[27] Raymond D, Peterson E (2011). A critical review of early-onset and late-onset preeclampsia. Obstet Gynecol Surv.

[28] Uzan J, Carbonnel M, Piconne O, Asmar R, Ayoubi JM (2011). Preeclampsia: pathophysiology, diagnosis, and management. Vasc Health Risk Manag.

[29] Chaiworapongsa T, Romero R, Espinoza J, Bujold E (2004). Evidence supporting a role for blockade of the vascular endothelial growth factor system in the pathophysiology of preeclampsia. Am J Obstet Gynecol.

[30] Lygnos MC, Pappa KI, Papadaki HA (2006). Changes in maternal plasma levels of VEGF, bFGF, TGF-beta1, ET-1 and sKL during uncomplicated pregnancy, hypertensive pregnancy and gestational diabetes. In Vivo.

[31] Sharkey AM, Cooper JC, Balmforth JR, Mclaren J (1996). Maternal plasma levels of vascular endothelial growth factor in normotensive pregnancies and in pregnancies complicated by preeclampsia. Euro J of Clin Invest.

[32] Yelumalai S, Muniandy S, Omar SZ, Qvist R (2010). Pregnancy induced hypertension and preeclampsia: Levels of angiogenic factors in Malaysian women. J Clin Biochem Nutr.

[33] Belgore FM, Lip GY, Blann AD (2000). Vascular endothelial growth factor and it receptor, flt-1 in smokers and non-smokers. Br J Biomed Sci.

